# Competition of Intra- and Intermolecular Forces in Anthraquinone and Its Selected Derivatives

**DOI:** 10.3390/molecules26113448

**Published:** 2021-06-06

**Authors:** Kamil Raczyński, Andrzej Pihut, Jarosław J. Panek, Aneta Jezierska

**Affiliations:** Faculty of Chemistry, University of Wrocław, ul. F. Joliot-Curie 14, 50-383 Wrocław, Poland; 298941@uwr.edu.pl (K.R.); andrzej.pihut@outlook.com (A.P.); jaroslaw.panek@chem.uni.wroc.pl (J.J.P.)

**Keywords:** anthraquinone, DFT, MP2, AIM, ELF, SAPT, TD-DFT, CPMD

## Abstract

Intra- and intermolecular forces competition was investigated in the 9,10-anthraquinone (**1**) and its derivatives both in vacuo and in the crystalline phase. The 1,8-dihydroxy-9,10-anthraquinone (**2**) and 1,8-dinitro-4,5-dihydroxy-anthraquinone (**3**) contain Resonance-Assisted Hydrogen Bonds (RAHBs). The intramolecular hydrogen bonds properties were studied in the electronic ground and excited states employing Møller-Plesset second-order perturbation theory (MP2), Density Functional Theory (DFT) method in its classical formulation as well as its time-dependent extension (TD-DFT). The proton potential functions were obtained via scanning the OH distance and the dihedral angle related to the OH group rotation. The topological analysis was carried out on the basis of theories of Atoms in Molecules (AIM—molecular topology, properties of critical points, AIM charges) and Electron Localization Function (ELF—2D maps showing bonding patterns, calculation of electron populations in the hydrogen bonds). The Symmetry-Adapted Perturbation Theory (SAPT) was applied for the energy decomposition in the dimers. Finally, Car–Parrinello molecular dynamics (CPMD) simulations were performed to shed light onto bridge protons dynamics upon environmental influence. The vibrational features of the OH stretching were revealed using Fourier transformation of the autocorrelation function of atomic velocity. It was found that the presence of OH and NO${}_{2}$ substituents influenced the geometric and electronic structure of the anthraquinone moiety. The AIM and ELF analyses showed that the quantitative differences between hydrogen bonds properties could be neglected. The bridged protons are localized on the donor side in the electronic ground state, but the Excited-State Intramolecular Proton Transfer (ESIPT) was noticed as a result of the TD-DFT calculations. The hierarchy of interactions determined by SAPT method indicated that weak hydrogen bonds play modifying role in the organization of these crystal structures, but primary ordering factor is dispersion. The CPMD crystalline phase results indicated bridged proton-sharing in the compound **2**.

## 1. Introduction

Intermolecular non-covalent forces of diverse types (from weak dispersion to strong electrostatics) are as important for understanding of condensed phases as covalent bond is for molecules [1,2,3,4,5]. In fact, both intra- and intermolecular forces are necessary for complete description of chemical systems. Current research trends put more and more emphasis on the factors governing formation of supramolecular systems [6,7]. Scientists are approaching the level of predictive design, moving from observation towards successful strategies of obtaining desired structural features [8]. Our quest to shed more light onto self-assembly of molecules, and reveal how atoms and molecules form larger structures at nanoscale, requires detailed description of intra- and intermolecular forces. Especially interesting cases are those in which these types of forces compete, and the final outcome is a result of a delicate balance [9,10,11,12,13]. Herein, we present a study of anthraquinone derivatives in which diverse types of intermolecular forces are affected by intramolecular hydrogen bonding and substituent effects.

**Figure 1:**
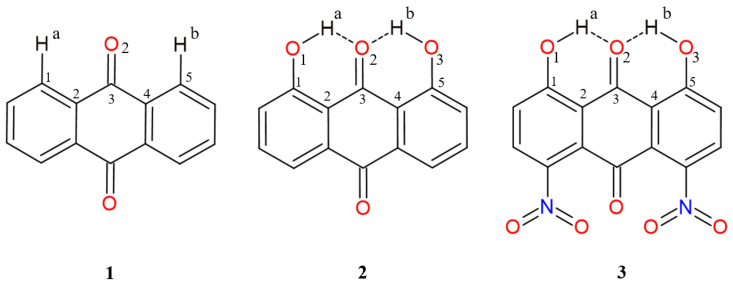
The structures of the studied anthraquinones: 9,10-anthraquinone (**1**), 1,8-dihydroxy-9,10-anthraquinone (**2**) and 1,8-dinitro-4,5-dihydroxy-anthraquinone (**3**) with atoms numbering scheme prepared for the study. Only atoms of interest are marked. The atom coloring scheme is as follows: grey—carbon, red—oxygen, blue—nitrogen and white—hydrogen. The dotted line indicates the presence of intramolecular hydrogen bonds.

Anthraquinone (a common name for 9,10-anthraquinone) is a three-ring compound resulting from anthracene oxidation (see Figure 1, compound **1**). The aromaticity of the middle ring is strongly limited, as usual for quinones, because of the presence of carbonyl functions [14]. The presence of aromatic skeleton makes this molecule, and its derivatives, rather rigid structurally, which can be important and promising for the design of well-defined larger structures. On the other hand, aromatic systems undergo substitution reactions relatively easily, so that influence of inductive and steric effects can be introduced into the molecule. This makes the substituents the driving forces of molecular organization at the microscopic level, which adds to the value of anthraquinones as building blocks.

The group of anthraquinones is believed to be the largest family of naturally occurring quinone derivatives. Anthraquinone and its derivatives found some practical application as, e.g., digester additive in production of paper pulp by alkaline processes [15] or as an electrolyte in flow battery which can provide long term electrical storage [16]. Additionally, anthraquinone is a well-known building block of many dyes, which could be divided into natural and synthetic [17]. In connection with the fact mentioned above that this family is the most numerous among natural quinones, it is necessary to mention that over 700 compounds of the anthraquinone group were identified as natural dyes (over 200 from plants, the rest from lichens and fungi) [18]. An interesting example is alizarin—red dye (1,2-dihydroxyanthraquinone), the first natural dye, which has been produced synthetically since 1869 [19]. The characteristics provided above make these molecules very attractive for synthetic organic chemists, striving to attain total synthesis of more and more representatives of this class of compounds. The continuous interest in this family of chemical species is highlighted by usage statistics of the term “anthraquinone” as a publication topic: the Web of Science reports 356 such publications in this year alone (from January to May 2021), and 3342 papers within the last 5 years.

**Figure 2:**
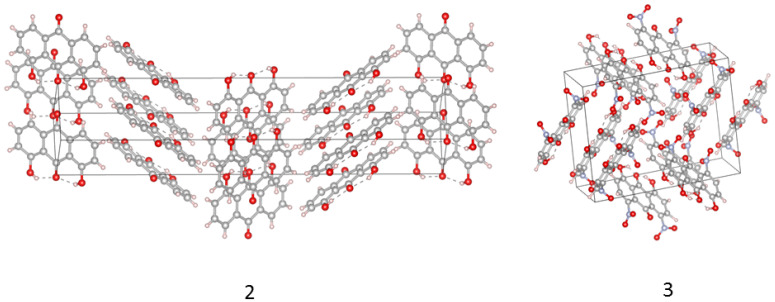
The crystallographic unit cells with the molecules arrangement for the compounds **2** and **3** [21,22]. The dotted lines indicate the presence of intramolecular hydrogen bonds. The data was used for CPMD simulations in the crystalline phase.

In the current study, we present quantum-chemical results for 9,10-anthraquinone (**1**) [20] and its two derivatives (1,8-dihydroxy-9,10-anthraquinone (**2**) [21] and 1,8-dinitro-4,5-dihydroxy-anthraquinone (**3**) [22]), (see Figure 1 and Figure S1). The chemical composition of anthraquinone (rigid part consisting of three fused rings) makes the molecule an interesting object for structure modification based on various substituents. In our case, the anthraquinone was treated as a reference to demonstrate differences in geometric and electronic structure as well as self-assembly of molecules substituted by OH and NO${}_{2}$ groups. Special attention was paid to intra- and intermolecular interactions responsible for the structure stabilization and molecules arrangement in the crystal unit cell (see Figure 2 where hydrogen bonded derivatives are presented).

The introduction of the OH groups as substituents resulted in intramolecular hydrogen bonds formation in the compounds **2** and **3**. The appearance of the substituents resulted in the inductive effects influence on the electronic structure of the anthraquinone moiety. The choice of nitro groups was dictated by their strong substituent influence, measured by diverse parameters including classical Hammett constants [23]. In addition, the quasi-rings were formed. The hydrogen bonding is classified among the strongest non-covalent interactions [24]. In the compounds there are so called Resonance-Assisted Hydrogen Bonds (RAHBs) [25]. The presence of intramolecular hydrogen bonding could be associated with various phenomena, e.g., proton transfer from the donor to the acceptor atom. The strength of the bonding could be further modified by steric and inductive effects [26]. The presence of the hydrogen bonding is visible as well in the spectroscopy, e.g., in the Nuclear Magnetic Resonance (NMR), strong hydrogen bonds are revealed by downfield shifts in the ${}^{1}$H NMR spectrum. In the infrared spectroscopy (IR), the hydrogen bonding shifts the X-H stretching frequency to lower energy (the decrease in the vibration frequency is observed) [27,28,29].

The common names for 1,8-dihydroxy-9,10-anthraquinone (**2**) are dantron and chrysazin. The main uses of **2** are as medical laxative (contemporarily in decline, because of suspected carcinogenicity) and as an intermediate for various dyes. Our attention was drawn to this compound because **2** forms five polymorphs [21] (the structure assumed in our study is polymorph 5, see Figure 2). This shows how delicate the balance is between various factors governing the self-assembly and ultimate crystal structure of **2**. As we describe below, the full spectrum of interactions (dispersion, electrostatics, weak C-H···O bonds) should be taken into account.

Discussing further the chemical structure of the chosen compounds, we have to underline that in the compound **3** the NO${}_{2}$ groups were added to the 1,8-dihydroxy-9,10-anthraquinone. Therefore, when describing the substituent effects, the molecule could be divided into three parts: (i) substituent X (in our case the NO${}_{2}$ groups); (ii) functional group Y where the effect is investigated “reaction center” (in our case OH groups); (iii) “transmitter” R (in our case anthraquinone moiety). The schematic presentation of the structure division is presented in the Supplementary Information (see Figure S2). Such division has been reported while investigating the physical interpretation of inductive and resonance substituent effects [30].

Compound **3** is not commercially significant, but it is a potential colorant. Its crystals exhibit pleochromism (different coloring when viewing at different angles), and its synthesis was undertaken to study the impact of loss of planarity on the anthraquinone derivatives [22]. In our case, introduction of diverse interaction centers (nitro groups as strongly acting substituents and hydrogen bond acceptors) into the three-ring skeleton was the main motivation to select this compound for deeper study.

The main aim of the study was devoted to the description of intra- and intermolecular forces present in the studied set of anthraquinone-type compounds. In order to achieve this goal, we have employed several theoretical approaches. The simulations were performed in the electronic ground and excited states as well as in vacuo and crystalline phase. We have analyzed monomeric, dimeric and crystal forms of the investigated compounds. In particular, we paid special attention to: (i) the metric paramteres changes upon substituents introduction to the anthraquinone moiety (ii) the electronic structure changes as a result of the presence of intramolecular hydrogen bonds; (iii) the molecular topology study on the basis of Atoms in Molecules (AIM) [31] and Electron Localization Function (ELF) [32]; (iv) intermolecular forces present in the studied dimers using Symmetry-Adapted Perturbation Theory (SAPT) method [33]; (v) proton potential functions in the electronic ground and excited states with special emphasis of Excited-State Intramolecular Proton Transfer (ESIPT) present in the molecules; (vi) bridged protons dynamics in vacuo and crystalline phase; (vii) spectroscopic properties reproduced by the Fourier transformation of the time autocorrelation function of atomic velocity.

Herein, we present the description of transition from single molecules through dimers to crystals taking into account various forces responsible for the self-assembly of the discussed in the study anthraquinones.

## 2. Results and Discussion

The competition of intra- and intermolecular forces was analyzed on the basis of quantum-chemical simulations performed in vacuo and in the crystalline phase for the studied anthraquinones. The monomeric, dimeric forms and finally the crystal structure were taken into account and analyzed in detail.

### 2.1. Geometric Parameters for Monomeric Forms and Proton Potential Functions

Thorough discussion of computational results should include at the beginning an important task of estimation how the applied levels of theory reproduce the experimental geometrical parameters. The extensive discussion of the performance of applied levels of theory with respect to the X-ray data [20,21,22] of the compounds **1**–**3** is given in the Supplementary Information. As it is shown in Tables S1–S3, the computationally obtained results correspond well with the experimental findings. Special attention was paid to the properties of intramolecular hydrogen bonds. They are classified as Resonance-Assisted Hydrogen bonds (RAHB) and their presence introduced so-called quasi-rings and reduced the mobility of the OH groups. The quasi-ring formation usually is associated with the internal geometric parameters as well as electronic structure reorganization. Therefore, we analyzed using different perspectives the substituents effect on the anthraquinone moiety.

**Figure 3:**
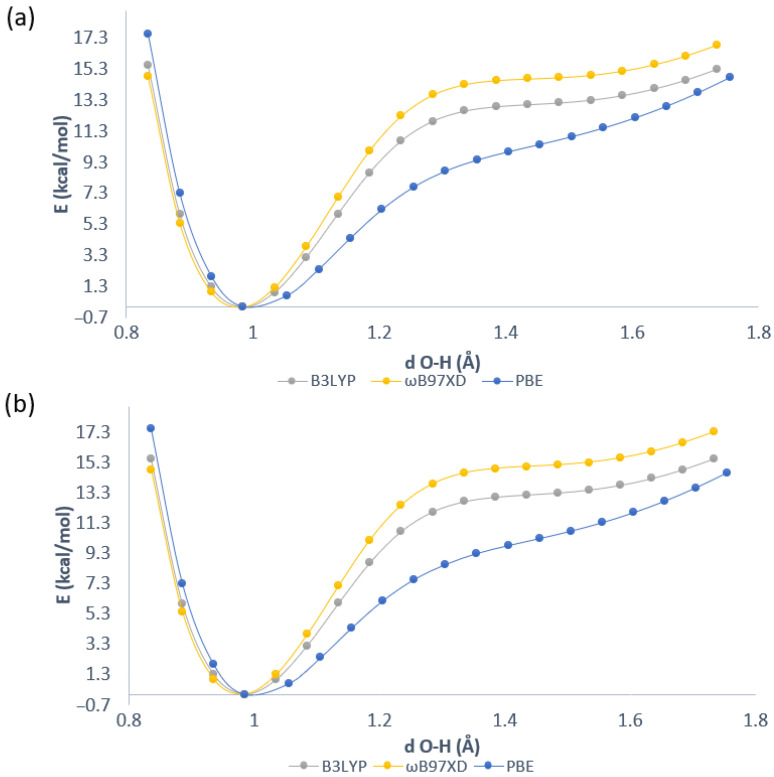
The potential energy scans for the proton motion in the hydrogen bridges of (**a**) 1,8-dihydroxy-9,10-anthraquinone (**2**) and (**b**) 1,8-dinitro-4,5-dihydroxy-anthraquinone (**3**). X axis: donor-proton ($O1-H{}^{a}$) distance.

The proton reaction path was studied for one of intramolecular hydrogen bonds in compounds **2** and **3**. The results of the analyses are presented in Figure 3. The simulations were performed at the DFT level of theory with application of 6-311+G(d,p) basis set. One energy minimum was obtained in case of both compounds. We can conclude that the proton transfer phenomenon is not preferable in both studied anthraquinones. The substituent effects in the compound **3** are subtle and they did not affect proton potential functions. The lack of secondary, acceptor-side minimum means that the proton potential functions do not possess classically defined barriers; however, we will use this term to indicate the expense of energy necessary to promote the proton to the wide plateau at the acceptor side. The energy barrier visible in Figure 3 ranges from ca. 12.5 kcal/mol to 15 kcal/mol depending on the functional. The lowest energy barrier was obtained for the PBE functional in both compounds while the highest was for the ωB97XD functional. Summarizing, in the electronic ground state, the bridged proton is localized on the donor side according to the simulations performed in vacuo where the molecules possess all degrees of freedom and the environmental effects as well as the intermolecular interactions were not included. In the next paragraph, the topological analyses results are presented to discuss the bonding pattern and electronic structure reorganization as a consequence of intramolecular hydrogen bonds formation and the presence of the NO${}_{2}$ substituent.

### 2.2. Topological Analysis of Molecular Scalar Fields—A Tool to Reveal the Pattern of Bonding

#### 2.2.1. Atoms in Molecules (AIM)

The topological analysis was performed for structures in equilibrium. The wavefunctions for the AIM analysis were obtained at the B3LYP/6-311+G(d) level of theory. The net atomic charges values for atoms of interest are presented in Table 1 (for clarity see Figure 1). The 9,10-anthraquinone served as a reference to detect differences introduced by intramolecular hydrogen bonds and N$O_{2}$ groups as additional substituents in the anthraquinone moiety. The net atomic charge of bridged protons is equal ca. 0.6 [e] in compounds **2** and **3**, respectively. The values of net atomic charges of O1 and O3 atoms derived from O-H groups are equal ca. −1.0945 [e] and −1.0869 [e]. The net charge value of atom O2 differs between 9,10-anthraquinone and its hydrogen-bonded derivatives. The presence of the intramolecular hydrogen bonds decreased the values ca. 0.0394 [e] and 0.0314 [e] in the compounds **2** and **3**, respectively. Significant changes in electron density were noticed for C1 and C5 atoms. The introduction of O-H groups as substituents affected the net charge values as it is presented in Table 1. The net atomic charge values of C2 and C4 of 9,10-anthraquinone are lower than in the compounds **2** and **3**. Therefore, the substituent effects’ influence on the electron density were noticed. A similar conclusion could be drawn for the C3 atom, but the net charge value is the highest in 9,10-anthraquinone. The electronic structure analysis showed quantitative differences introduced by substituents and intramolecular forces to the anthraquinone moiety.

**Figure 4:**
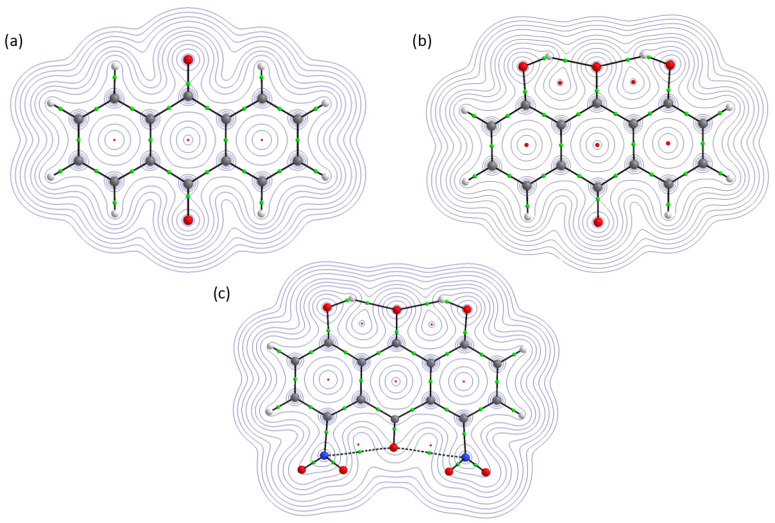
Electron density contour maps of the investigated molecules: (**a**) Compound **1**, (**b**) Compound **2**, (**c**) Compound **3**. Small green spheres denote Bond Critical Points, while small red spheres denote Ring Critical Points.

The electron density maps for the studied compounds are presented in Figure 4. They are in agreement with the conventional view of intramolecular interactions. The topology of 9,10-anthraquinone was characterized on the basis of Bond and Ring Critical Points (BCPs and RCPs). They are BCPs related to the covalent bonding present in the molecule. In addition, three RCPs were detected confirming the structure composition of the molecule. Again, the unsubstituted anthraquinone served as a reference to show differences in the studied molecules due to the additional intramolecular forces. The presence of intramolecular hydrogen bonds was confirmed by BCPs found between the bridged protons and the acceptor atoms in compounds **2** and **3**. In case of the compound **3**, BCPs were noticed between oxygen atom from the athraquinone moiety and nitrogen from the NO${}_{2}$ groups. This could be a result of a sterically driven electron densities overlap and does not indicate important intramolecular interactions. The presence of intramolecular hydrogen bonds resulted in quasi-rings formation in compounds **2** and **3**. Therefore, the compound **2** contains five RCPs—three derived from the anthraquinone moiety and two from the quasi-rings. The AIM molecular graph of the compound **3** shows seven RCPs. However, two of them (where NO${}_{2}$ groups are contributing) do not belong to quasi-rings and they are a result of electron density distribution driven by steric effects introduced by the substituents. The quantitative estimation of electron density and its Laplacian at Bond Critical Points (BCPs) is presented in Table 2.

The quantitative description given by the values indicated small differences in the electron density and its Laplacian introduced to the anthraquinone moiety by the presence of intramolecular hydrogen bonds and NO${}_{2}$ groups. The hydrogen bond electron density and its Laplacian at BCPs in both compounds have positive values, which is in agreement with Popelier’s criteria for hydrogen bonding [34]. The values of electron density at BCPs of hydrogen bonds are equal in both compounds; however, there is a difference in the Laplacian, which is lower for the compound **2**. The electron density and its Laplacian at $H^{a}$-O1 and O3-$H^{b}$ at BCPs is smaller in the compound **3** than in the compound **2**. The noticeable difference could be an effect of the presence of the NO${}_{2}$ groups. Comparing 9,10-anthraquinone with its substituted derivatives, one can see that electron density at C1-C2 and C4-C5 BCPs is larger than in the compounds **2** and **3**, but its Laplacian is smaller. An opposite situation was found for electron density and its Laplacian at C2-C3 and C3-C4 BCPs. Comparing further the electron density and its Laplacian values at BCPs related to the C1-C2, C2-C3, C3-C4 and C4-C5, it was noticed that for the compound **2** the electron density value is larger, but its Laplacian is smaller than in the compound **3**.

The Ring Critical Points (RCPs) values are presented in Table S4. Let us look at 9,10-anthraquinone electron density and its values at the RCPs. The values of electron density and its Laplacian are lower in the ring II comparing to the rings I and III. A similar tendency was observed for the compounds **2** and **3**, respectively. However, the quasi-rings formation had an influence on the RCPs in the compounds **2** and **3**—the electron density and its Laplacian values are smaller for rings I and III comparing to the compound **1**. In the ring II, an opposite situation was found (see Table S4 for details). As it was shown, the electron density and its Laplacian at RCPs in the quasi-rings are quantitatively equal. It was indicated as well that the presence of NO${}_{2}$ groups in the anthraquinone moiety introduced changes in the electronic structure, which were estimated quantitatively by the AIM theory application in the current study.

#### 2.2.2. Electron Localization Function (ELF)

**Figure 5:**
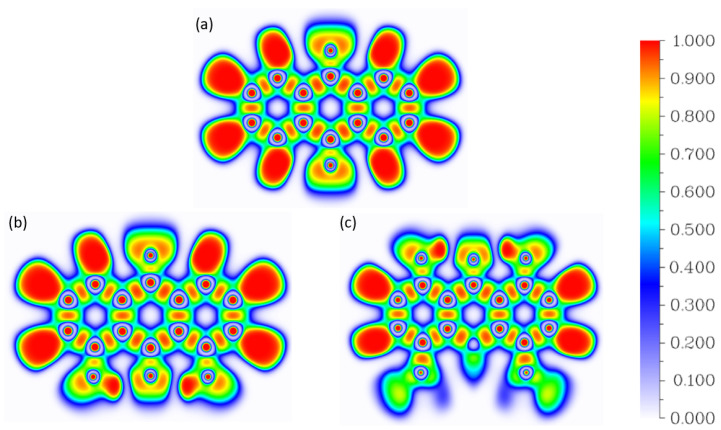
2D maps of Electron Localization Function (ELF) for the studied compounds (**a**) **1**, (**b**) **2** and (**c**) **3** codified by colors in a range form 0 (purple) to 1 (red). The ELF was calculated at the B3LYP/6-311+G(d) level of theory.

In general, the ELF basins correspond to the regions with well-defined chemical role. In particular, the cross-sections of the ELF field (Figure 5) show the atomic cores (as red dots indicating strong electron pairing inside the 1s${}^{2}$ cores) connected with the valence (bonding) regions (elongated orange ovals) and lone pairs. The case of hydrogen atoms is special—lacking cores, these atoms form large valence domains. The cross-sections in the molecular planes are valuable because they depict in great detail the bonding schemes around the quinone oxygen atoms, and the hydrogen bridges. However, 3D representations depicted in Figure S4 are able to show more details of the aromatic system and the out-of-plane nitro groups.

The vicinity of the hydrogen bridge in the studied compounds has the following composition in the ELF framework: the donor O1 atom has its lone pair region Lp(O1), and the acceptor O2 atom exhibits two symmetrical lone pairs Lp(O2). The bridge itself corresponds to the valence V(O1-H${}^{a}$) basin. Compound **1**, having no hydrogen bridges, posses only the two symmetrical Lp(O2) regions. All the electron populations inside these basins are reported in Table 3.

The Lp(O2) electron populations are very sensitive to the presence of the hydrogen bonding. An increase in the electron population of the O2 acceptor atom lone pairs is related to the polarization of the acceptor by the bridged proton—the magnitude of this polarization (difference between Lp(O2) population for **1** and either **2** or **3**) is 0.11 e, and it is not affected by the presence of nitro substituents in **3**. Note, however, that the lone pair domains of O2 contain more than one electron pair (populations larger than 2 e), in agreement with the strong electronegativity and electron-withdrawing properties of the oxygen atom. The same can be said about the lone pairs of the O1 donor; these are merged into one Lp(O1) region with population larger than 4 e. A small but visible difference of 0.04 e between the Lp(O1) populations in **2** and **3** can be potentially associated with the presence of electron-withdrawing nitro substituents in **3**, competing with O1 for the electron density. Finally, the donor–proton bonds, formally single, are predicted to be weakened (populations less than 2 e), in agreement with the electronegativity difference. For comparison, the ELF basins corresponding to the C-H bonds have electron populations of 2.12–2.14 e. Thus, the ELF analysis shows the subtle effects of the nitro substituents, and presents the extent of the charge transfer due to electronegativity of the oxygen atoms.

### 2.3. Intermolecular Forces in the Crystal Lattice: A Symmetry-Adapted Perturbation Theory Perspective

**Figure 6:**
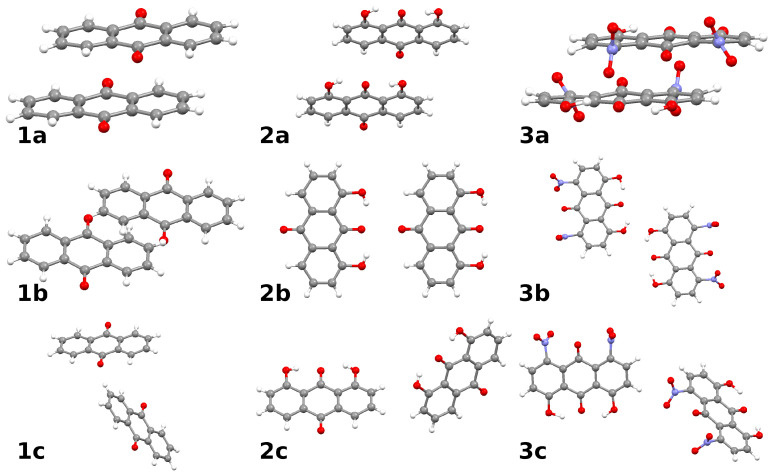
The structures of the dimers of compounds **1**, **2**, and **3** taken into account in the interaction energy study. Additional letter labels indicate: **a**—stacked dimers, **b**—head-to-head or head-to-tail structures, **c**—structures with C-H···O bonding. Atoms coloring scheme: grey—carbon, red—oxygen, blue—nitrogen, white—hydrogen.

The crystal structures of the three studied compounds [20,21,22] share some similarities, but differ in some important details. In particular, a given molecule in the lattice forms at least three types of distinct contacts with its neighbors. We have extracted the corresponding dimers from the crystal structures, see Figure 6, and identified the following:

- Typical stacked structures, labeled as dimers **1a**, **2a**, **3a**—note that, because of the substituents, the monomers in **2a** and **3a** are not placed directly in vertical alignment;
- Head-to-head or head-to-tail structures **1b**, **2b**, **3b**, where **2b** is head-to-tail, **3b** is head-to-head, and **1b** is less typical because one of the interacting molecules is raised by half of the interplanar stacking separation of **1a**;
- Various forms of the C-H···O interactions: in **1c** the C-H bond targets the carbonyl oxygen atom, while in **2c** and **3c** there are lateral C-H···O bonds, respectively, to the hydroxyl group or to the nitro group of the neighboring molecule.

The dimers differ not only in their structures, but also in the origin of the intermolecular bonding, revealed in the detailed partitioning of the interaction energies. Results of such calculations are compiled in Table 4 and divided into the four principal groups: electrostatic (without the effect of polarization by the neighbor), exchange (which is the short-range Pauli repulsion), induction (which is based on mutual polarization of the monomers), and dispersion. It is very interesting to see that the presence of substituents in fact does not warrant stronger intermolecular forces—the largest magnitude of interaction is calculated for the dimer **1a** of 9,10-anthraquinone. Before delving into details, we note that this unexpected finding comes, with all probability, from the fact that the introduced hydroxyl substituents are engaged in the intramolecular contacts and do not contribute to the intermolecular binding, whereas the nitro groups of **3** are bulky and can enforce suboptimal stacking arrangement.

A detailed analysis of Table 4 shows that the least important factor of the dimer stability is induction (polarization). This stems from the lack of large, “soft” electron clouds—while the aromatic system contains strongly delocalized orbitals, these are at the same time confined by the molecular skeleton and are unable to provide large induction energy. Note also that the electrostatic contribution is quite significant for the compound **1**, which—at a first glance, due to the symmetry of the molecular skeleton—should be much less polar than the compounds **2** and **3** possessing polar hydroxyl substituents. Our earlier experience with aromatic systems of high symmetry [36] indicates that quadrupole–quadrupole interactions are significant sources of interaction strength for symmetric molecules of this kind, and this work shows that these interactions in **1** can compete in strength with dipole–dipole forces in **2** and **3**. Interestingly, the dimer **2b** is arranged in a head-to-tail manner, which is less optimal (electrostatic energy of −0.96 kcal/mol) for the permanent dipole interactions than the head-to-head arrangement found in **3b** (−4.25 kcal/mol electrostatic term). All these phenomena, however, are overwhelmed by the dispersion contribution in the stacked dimers, resulting from the delocalized electron clouds. The role of dispersion is so large that the total interaction energy calculated at the simplified SAPT0 level is predicted to be smaller than the full SAPT2 value, contrary to usual findings. This fact is most pronounced for the stacked dimers **1a**–**3a**. A large area of aromatic systems makes the dispersion stronger than specific C-H···O interactions of the dimers **1c**–**3c**. Each dimer exhibits only one such weak hydrogen bond, and - in agreement with the other types of dimers—it is weakest in the 1,8-dihydroxy-9,10-anthraquinone **2**.

The hierarchy of interactions determined in this section shows that the weak hydrogen bonds play modifying role in the organization of these crystal structures, but primary ordering factor is dispersion.

### 2.4. Proton Potential Functions in the Electronic Ground and Excited States

**Figure 7:**
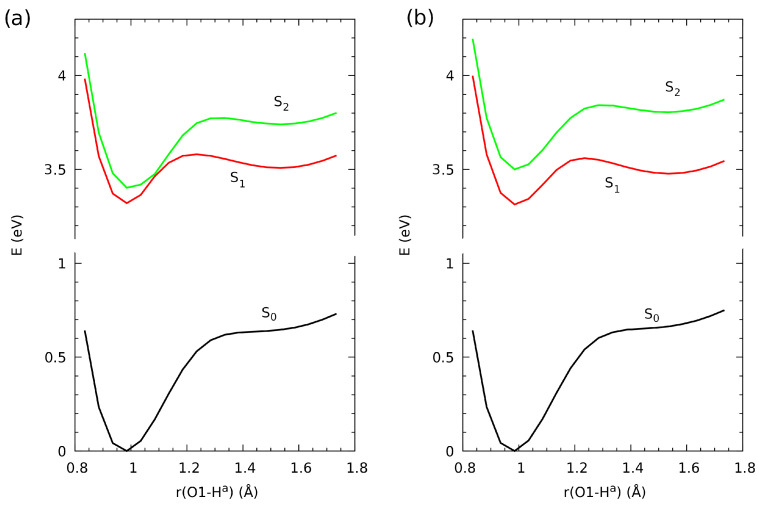
The proton potential functions for the proton transfer pathway in the ground electronic state and two lowest-lying excited singlet states from TD-DFT calculations at the ωB97XD/6-311+G(d,p) level. (**a**) Results for compound **2**, 1,8-dihydroxy-9,10-anthraquinone. (**b**) Results for compound **3**, 1,8-dinitro-4,5-dihydroxyanthraquinone.

Properties of the hydrogen bonds in aromatic systems can be modified by such factors as substituents, number of rings, or electronic excitation. The latter is particularly interesting as it does not require modification of the molecular structure, and only the irradiation is necessary. For this reason, we have investigated the proton potential functions for the studied systems (compounds **2** and **3**) in three electronic singlet states, $S_{0}$, $S_{1}$ and $S_{2}$. The H${}^{a}$ proton pathways along the O1···O2 bridges, resulting from the optimized scan procedure (see Section 3.1 for details), are presented in Figure 7.

The ground state proton potential functions do not possess additional minima on the acceptor side; inflexion points at ca. 1.5 Å are present instead. This excludes the possibility of any long-lived proton transfer phenomenon, in agreement with the fact that such proton transfer would result in severe loss of aromaticity in the outermost aromatic rings. The inflexion points, located 15 kcal/mol above the minimum, are not easily accessible. The situation changes dramatically when the molecules are subject to UV/Vis irradiation. The PES for $S_{1}$ and $S_{2}$ possess minima not only at the donor side, but also at the acceptor side. The acceptor-side minima for the $S_{1}$ excited state are 5 kcal/mol above the donor-side structure, and the barrier height is 6.5 kcal/mol (the values are very similar for both compounds **2** and **3**). This enables the possibility of temporary, short-lived Excited-State Intramolecular Proton Transfer (ESIPT) phenomenon. On the other hand, the acceptor-side minima on the $S_{2}$ excited state PES are less suited for the ESIPT—they are shallower and lie somewhat higher in relation to the donor-side structures. The most important difference between the $S_{1}$ and $S_{2}$ excited state PES for compounds **2** and **3** is that for **2**, the two curves are very close (almost degenerate) at the O1-$H^{a}$ distance of 1.1 Å, but for **3**, the two states are always well separated. This fact must be attributed to the substituent effects of the nitro groups in **3**.

**Figure 8:**
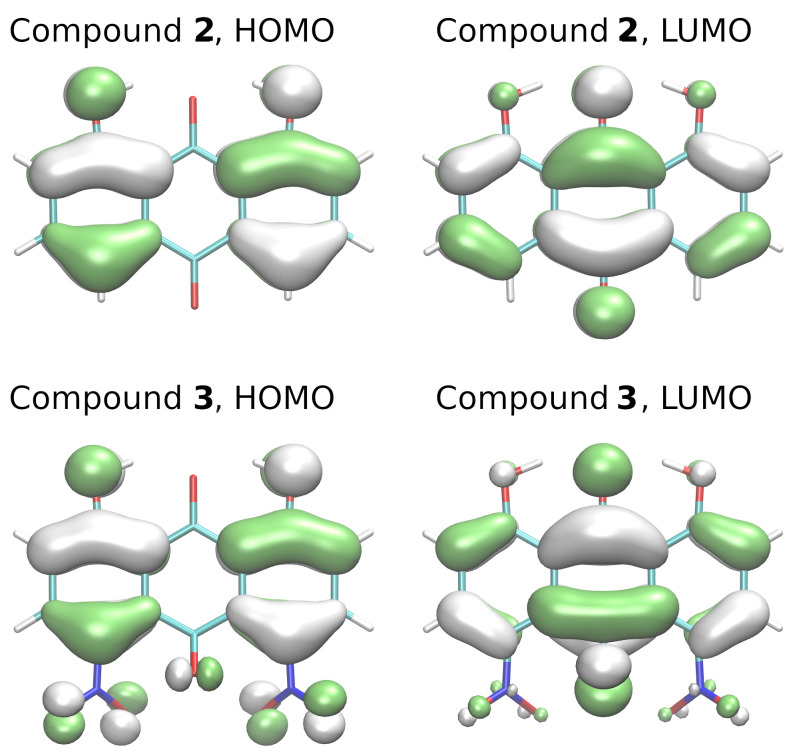
HOMO and LUMO orbitals for the compounds **2** and **3** computed at the ωB97XD/6-311+G(d,p) level.

Shapes of the S${}_{0}$ ground state highest occupied (HOMO) and the lowest unoccupied (LUMO) molecular orbitals have been frequently used to rationalize the properties of the lowest-lying excited states. The HOMO and LUMO orbitals for compounds **2** and **3** are presented in Figure 8. These orbitals have π character with respect to the plane of the aromatic system; therefore, under each feature shown in Figure 8, there is a corresponding feature of the opposite sign. It can be seen that while the orbitals are mostly delocalized over the rings, there is a significant contribution of HOMO orbitals on the donor O1 and O3 atoms. On the other hand, the LUMO is encompassing not the donor atoms, but the O2 proton acceptor. This agrees with the sensitivity of the proton potential function to the electronic excitation. The role of nitro substituents can be appreciated from the fact that the HOMO (and to a lesser extent, LUMO) of the compound **3** also involves these groups.

The fact that HOMO and LUMO encompass also the hydrogen bridge regions has prompted us to study the effect of the O1-H${}^{a}$ group rotation around the C1-O1 bond on the energy profiles of the ground state and S${}_{1}$, S${}_{2}$ excited states, in the spirit of a recent study by Cozza et al. [37]. The results, shown in Figure S5, indicate that the asymmetry introduced by non-equivalent positions of the -NO${}_{2}$ groups influences the height of the rotation energy barriers for the ground state energy profile of **3**. However, this effect is much less visible in the two excited states. Anthraquinone skeleton is an effective transmitter of the substituent influence, and—as we have already noted above—even relatively small variations of the angle of the nitro groups has noticeable impact on the energy profiles.

The reported shapes of the bridged proton potential energy surface (PES) for the electronic ground and excited states have distinct bearing on the dynamical behaviour of the compounds **2** and **3**. In particular, even if the proton transfer events in the ground state are not likely, significant anharmonicity of the O1-$H^{a}$ stretching mode is expected. The details will be revealed using the Car–Parrinello molecular dynamics (CPMD) approach.

### 2.5. Compounds ***2*** and ***3*** in Light of the Car–Parrinello Molecular Dynamics

**Figure 9:**
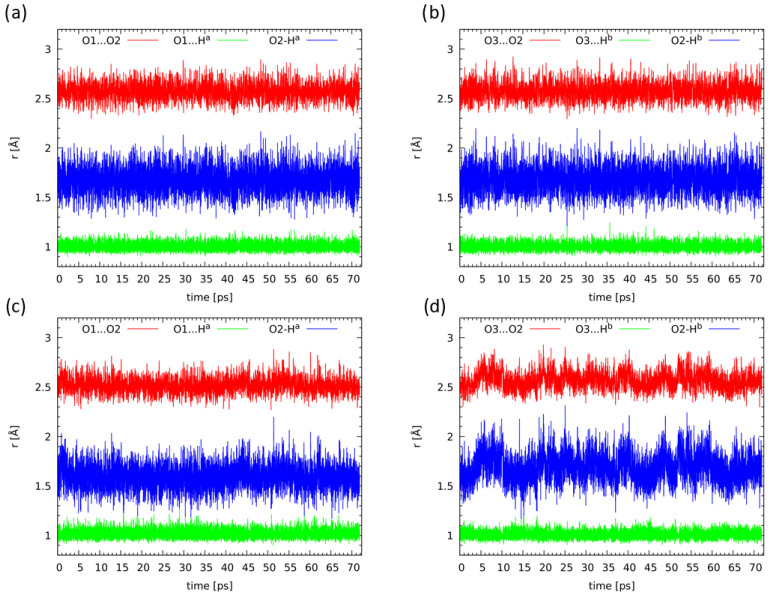
Time-evolution of interatomic distances of atoms involved in the intramolecular hydrogen bonds formation of the compound **2**, (**a**,**b**) results obtained from CPMD in vacuo while (**c**,**d**) in the crystalline phase.

Following gas phase findings for an isolated molecule of 9,10-anthraquinone and its derivatives using static MP2 and DFT methods, we have performed Car–Parrinello molecular dynamics (CPMD) simulations for the anthraquinones containing intramolecular hydrogen bonds. The simulations were carried out in vacuo and in the crystalline phase. The two-phases simulations enabled to make comparisons and show the external forces influence on, e.g., intramolecular hydrogen bond properties in our case. The hydrogen bridges dynamics of compounds **2** and **3** was analyzed in detail. As it is shown in Figure 9 where the CPMD results are presented for the compound **2**, the bridged protons are localized on the donor side during the whole simulations time. In the crystalline phase (lower part of Figure 9), proton-sharing events were noticed. The stronger proton mobility is associated with external forces influence on the hydrogen bridges dynamics, e.g., the presence of neighbouring molecules and crystal field. Concerning the compound **3**, the CPMD simulations gave very similar results in both applied phases and conditions. The bridged protons are localized on the donor side. However, we did not notice proton-sharing events. There is an interaction between an oxygen atom from the NO${}_{2}$ group of a neighbouring molecule with the bridged proton (see the crystal structure of the compound [22]). Therefore, there is a competition between intra- and intermolecular interactions. Most probably, that is the main reason why the bridged proton exhibited a smaller mobility comparing to the compound **2**. Moreover, the presence of the two NO${}_{2}$ groups introduces strong inductive and resonance effects into the aromatic rings, resulting in electron withdrawal from the aromatic rings. In the context of our simulations it must be noted that these effects are dependent on the orientation of the nitro group; compound **3** possesses non-equivalent arrangements of the nitro groups, thus modifying the properties of the hydrogen bridges. The crystal environment does not allow the orientation of the two nitro groups to become equalized, and this results in some differences in the calculated bridge properties. This is an important difference between the gas phase and solid state arrangement of various forces governing the molecular properties. The obtained results are presented in Figure S6. A detailed discussion of the time-evolution of metric parameters of atoms involved in the intramolecular hydrogen bonds formation is presented in the Supplementary Information.

**Figure 10:**
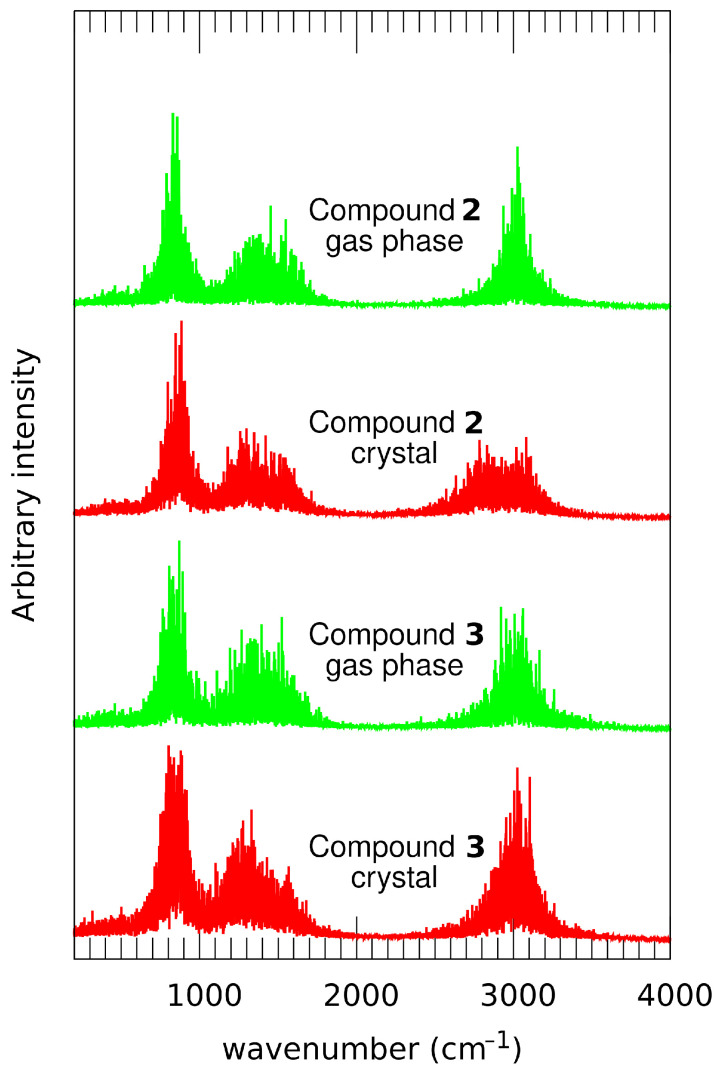
Vibrational features of the bridge protons in the studied compounds. Results of the gas phase and solid state CPMD simulations for compound **2**, 1,8-dihydroxy-9,10-anthraquinone, and compound **3**, 1,8-dinitro-4,5-dihydroxyanthraquinone.

The dynamics of the bridge protons are directly related to their vibrational signatures in the IR spectra. The CPMD approach allows for an easy decomposition of the vibrational power spectra into atomic contributions, but at the price of losing the information on the observable intensities. The intensities of atomic motions are registered instead, and the phenomenon of the increase in the stretching band intensity upon hydrogen bond formation is lost. For our purposes, however, it is more important to determine the contributions of the bridged protons to the vibrational spectrum. The results are presented in Figure 10. In agreement with the discussion on the structural parameters of the bridges presented above, the $\nu_{OH}$ bands for the compounds **2** and **3** are very similar. They are centered at 3000 cm${}^{-1}$ and extend from 2700 to 3300 cm${}^{-1}$. This indicates that the hydrogen bonds are medium-strong, but the protons are not strongly delocalized and stay at the donor side. The important exception is the solid state simulation of **2**, exhibiting more downshifted position of the band center (2930 cm${}^{-1}$) and the shape suggesting overlap of two vibrational regimes. This is in agreement with the instances of closer contacts of the bridged protons with the acceptor side revealed in the CPMD distance analysis—see Figure 9a,b. A survey of the SDBS spectral database [38] shows that the IR spectra of **1**, **2** and **3** have been measured. The compound **1** has no hydrogen bridges and thus it is the reference structure for the high wavenumber region. The compound **3** has an additional feature not related to the C-H bonds present also in **1**: a band of weak intensity, centered at 3050 cm${}^{-1}$. On the other hand, the IR spectrum of **2** has strong, broad feature extending practically from 1700 cm${}^{-1}$ upwards. Its center is hidden under the C-H modes, close to 2950 cm${}^{-1}$. Our CPMD results are in excellent agreement with these experimental data, revealing the unusual role of the nitro groups, which restrict the dynamics of the bridged protons. The spectroscopic evidence suggests that the hydrogen bonding is weaker, only middle-strong, than one could estimate from structural reasons (short O1···O2 interatomic distance corresponding to rather strong hydrogen bonds). The reason behind this interesting feature could be the fact that the small O1···O2 distance is enforced by the shape of the ring skeleton, and does not reflect the actual strength of the hydrogen bonds. Moreover, the proton transfer to the O2 acceptor atom would result in the reorganization of single and double bonds in the central ring, and this would conflict with the other carbonyl group. In this way, the “resonance assistance” phenomenon cannot be strong in the case of compounds **2** and **3**.

## 3. Computational Methodology

### 3.1. Static Electronic Ground State DFT and MP2 Models

The initial geometry for the quantum-chemical simulations of the studied anthraquinones monomers and dimers was taken from the crystallographic data deposited in the Cambridge Crystallographic Data Centre (CCDC) [39]. The CCDC code is 1031904 for 9,10-anthraquinone (**1**) [20], 719215 for 1,8-dihydroxy-9,10-anthraquinone [21] (**2**) and 1113560 for 1,8-dinitro-4,5-dihydroxy-anthraquinone (**3**) [22]), see Figure 1 and Figure 6. The geometry optimization of monomers was performed on the basis of Density Functional Theory (DFT) [40,41] and Møller–Plesset second-order perturbation theory (MP2) [42]. The DFT functionals denoted as B3LYP [43], PBE [44,45] and ωB97XD [46] with 6-311+G(d,p) triple-zeta valence split basis set [47,48] were applied to reproduce metric and electronic structure parameters. Additionally, the energy minimization was performed using MP2 method with assistance of the basis set mentioned above as a reference for the DFT computations. The harmonic frequencies were calculated to confirm that the obtained structures correspond with the minima on the Potential Energy Surface (PES). The proton reaction paths in the intramolecular hydrogen bonds were analyzed by means of the scan method with optimization. The OHO valence angle was fixed and the O-H distance was elongated with 0.05 Å increment while the remaining part of the molecules was optimized using all mentioned above functionals and basis set. The wavefunctions for further electronic structures analyses on the basis of Atoms and Molecules (AIM) [31] and Electron Localization Function (ELF) [32] theories were prepared using B3LYP/6-311+G(d) level of theory. This part of the simulations was performed with the Gaussian16 rev. C.01. suite of programs [49].

### 3.2. Electronic Structure and Topological Analyses on the Basis of Atoms in Molecules (AIM) and Electron Localization Function (ELF) Theories

The electronic structure analysis was performed using two methods: (i) Atoms in Molecules (AIM) [31] and (ii) Electron Localization Function (ELF) [32] for the structures in equilibrium of the studied anthraquinones.

The AIM theory served for electronic structure and topology study of the anthraquinones. Particular attention was paid to the intramolecular hydrogen bonds properties. The AIM atomic charges were computed for the whole molecules, but we have reported on values obtained for atoms involved in the hydrogen bonds formation as well as for carbon atoms associated with the quasi-rings. The electron density and its Laplacian at Bond and Ring Critical Points (BCPs and RCPs) were used as descriptors confirming the presence of the hydrogen bonds. Additionally, the substituent effects influence on the hydrogen bonds property and anthraquinone moiety were studied. The AIM analysis was carried out with the AIMAll package [50]. The ELF theory provides molecular space partitioning, which gives information of different bonding functionalities. This analysis is useful especially for molecules containing hydrogen bonding—to study consequences related to the proton position in the hydrogen bridge. There are core and valence basins. The first are related to the chemically inert electron density while the latter to the bonds and lone electron pairs. The ELF analysis was performed using the DGrid 5.1 [51] and Multiwfn [52] programs. The 3D visualization of ELF was performed with UCSF ChimeraX, developed by the Resource for Biocomputing, Visualization, and Informatics at the University of California, San Francisco, with support from National Institutes of Health R01-GM129325 and the Office of Cyber Infrastructure and Computational Biology, National Institute of Allergy and Infectious Diseases [53].

The AIM theory was found to be valuable in the interpretation of the charge density and its association with other chemical concepts (e.g., atoms, bonds etc.) [31]. The ELF theory describes chemical bonds with employment of rigorous partitioning of molecular space, being opposed to classical well-known concepts of chemical bonding [32]. It was developed using topological analysis of local quantum-mechanical functions connected with the Pauli’s exclusion principle where the local maxima specify the so-called “localization attractors”. Three basic types of the “localization attractors” were defined: bonding, non-bonding, and core. The bonding attractors describe the shared-electron interactions. The spatial arrangements of localization attractors gives a basis for a well defined bonds classification. An application of the two theories enabled a complete study of the bridged protons property and consequences related to the presence of the NO${}_{2}$ groups in the *para* position in respect to the OH groups.

### 3.3. Symmetry-Adapted Perturbation Theory (SAPT)

The energy decomposition of the dimers of the investigated anthraquinones (see Figure 6) was performed based on Symmetry-Adapted Perturbation Theory (SAPT) [33]. The energy was decomposed for dimers extracted from the X-ray data [20,21,22] in order to preserve geometries and reproduce intermolecular forces. The SAPT energy partitioning was carried out at the SAPT2 level of theory [35]. The interaction energy was calculated at the SAPT2/jun-cc-pVDZ level of theory (the jun-cc-pVDZ basis set, selected so that the dimers of compound **3** could be studied, corresponds to the modified aug-cc-pVDZ basis set where some diffuse functions are truncated [54,55]). The basis set superposition error (BSSE) correction [56] was included in the simulations of the dimers (the homodimers were divided into “monomers” in order to fulfil the requirements of the Boys–Bernardi method). The SAPT calculations were carried out using the Psi4 1.2.1 [57] program.

### 3.4. Time-Dependent Density Functional Theory (TD-DFT)

The Time-Dependent DFT calculations were carried out for compounds **2** and **3** using the Gussian16 rev. C.01. suite of programs [49]. The ωB97XD [46] functional with 6-311+G(d,p) triple-zeta valence split basis set [47,48] was employed to reproduce the proton transfer path in the electronic excited state. The geometries for the TD-DFT calculations were taken from the electronic ground state investigations of the proton reaction path (scan method, yielding 16 structures for each compound). Additionally, the O1-H${}^{a}$ group rotation around the C1-O1 bond was included as a separate potential energy scan, starting from the optimized structure and taking 24 steps of 15${}^{\circ }$ increment in the relevant dihedral angle. A single-point TD-DFT calculation encompassing 20 singlet states was carried out for each of the structures. The energy of two lowest-lying excited singlet states S${}_{1}$ and S${}_{2}$ was extracted from the calculation and used to construct proton potential functions in the excited electronic state. In order to support the TD-DFT study with insight from the S${}_{0}$ ground state, HOMO and LUMO orbitals in the ground state were computed for **2** and **3** at the ωB97XD/6-311+G(d,p) level of theory using the cubegen utility of the Gaussian16 suite, and visualized using the VMD 1.9.3 program [58].

### 3.5. Car–Parrinello Molecular Dynamics (CPMD) in the Gas Phase and Solid State

Car–Parrinello molecular dynamics (CPMD) simulations were performed in vacuo and in the crystalline phase. The intramolecular hydrogen bonds dynamics and spectroscopic signatures were developed for compounds **2** and **3**. The initial geometries for the CPMD runs were prepared based on crystal structures with (CCDC) code: 719215 for 1,8-dihydroxy-9,10-anthraquinone [21] (**2**) and 1113560 for 1,8-dinitro-4,5-dihydroxy-anthraquinone (**3**) [22], respectively. The isolated molecule models of compounds **2** and **3** are presented in Figure S1. The molecules were placed to cubic boxes with a = 14.5 Å for both compounds. The models for CPMD in the crystalline phase are presented in Figure 2 and Figure S3. The crystal unit cells served as a source of information for the proper models preparation. For the compound **2**, the experimental unit cell values are a = 5.7487 Å, b = 5.7487 Å, c = 31.438Å and β = $90^{\circ }$ with Z = 4 while for the compound **3** they are: a = 15.664 Å, b = 12.056 Å, c = 6.704 Å and α = $90^{\circ }$β = $94.20^{\circ }$γ = $90^{\circ }$ with Z = 4. The CPMD simulations were divided into three steps: geometry optimization, equilibration and production runs. The exchange correlation functional of Perdew–Burke–Ernzerhof denoted as PBE [44,45] and Troullier–Martins [59] norm-conserving pseudopotentials were applied. The fictitious electron mass (EMASS) was equal 400 a.u. for both compounds during the gas phase and solid state simulations. The time-step was set to 3 a.u. and the kinetic energy cutoff for the plane-wave basis set was 100 Ry. The CPMD simulations were performed at 297 K temperature controlled by Nosé–Hoover thermostat [60,61]. The empirical van der Waals corrections proposed by Grimme [62] were added to reproduce the intermolecular weak forces. The translational and rotational movements were removed from the CPMD data collection in the gas phase. The Hockney’s scheme [63] was applied in order to remove the interactions with periodic images of the cubic cells. Additionally, the solid state CPMD was performed with Γ point approximation [64] and Periodic Boundary Conditions (PBCs). The real-space electrostatic summations was set to TESR = 8 nearest neighbours in each direction. The initial part of the simulations was taken as an equilibration (ca. 10,000 steps) and it was excluded during the data analyses. The CPMD trajectories were collected for 70 ps for both compounds in both phases. The post-processing procedures focused mostly on the intramolecular hydrogen bonds dynamics. The time-evolution of interatomic distances of atoms involved in the intramolecular hydrogen bond formation was analyzed. The spectroscopic signatures of the studied anthraquinones were reproduced based on power spectra of atomic velocity. The CPMD simulations were carried out with the CPMD 3.17.1 program [65]. The data was analyzed using the VMD 1.9.3 [58] program and home-made scripts. The graphical presentation of the obtained results in the current study was prepared with assistance of the VMD 1.9.3 [58], Vesta Ver.3.5.6 [66], and Gnuplot [67] programs.

## 4. Conclusions

This study highlights the case of the use of molecular symmetry to influence the substituent effects. The compounds **2** and **3** differ by the presence of two nitro substituents in **3**, placed in such a way that their effects on the intramolecular hydrogen bonds are counteracting each other. The presence of these substituents is visible in the structural parameters, and the non-planar orientation of the nitro groups influences the resonance and steric effects, modifying the hydrogen bridges. Quantum-chemical DFT and MP2 calculations have provided structures in an overall good agreement with the experiment. Introduction of substituents did not significantly change the physico-chemical characteristics of the anthraquinone moiety; therefore, we can predict that introduction of other substituents from diverse regions of the Hammett constant spectrum will not affect heavily the molecular structure of this class of compounds.

The proton potential surface calculations have revealed that the electronic ground state prefers location of the proton at the donor side, while two analyzed excited states possess secondary minima at the acceptor side, thus making possible the ESIPT phenomenon. Topological analyses (AIM, ELF) revealed that the presence of the hydrogen bonding affects strongly the electronic structure of the molecular fragments common for the **1**–**3** anthraquinones, and the effect of nitro substituents, however subtle, is also visible in the monomers. On the other hand, it is evident that the nitro groups enhance the possibility of intermolecular contacts and improve the magnitudes of the interaction energies in the dimers. The unexpected finding of this study is that the compound **1**, without substituents, forms stronger intermolecular contacts than the hydroxyl-bearing derivatives **2** and **3**. The intramolecular contacts seem to have preference over the intermolecular forces for these compounds. We have also determined that the dimers of the studied compounds, taken from their crystal structures, are held mostly by dispersion forces, while electrostatic quadrupole–quadrupole (for **1**) and other multipole moments are present, but less significant. These factors influence directly the details of self-assembly of larger structures for these compounds. The main structure-forming role of the skeleton constituted by three rigid, fused rings leads to formation of stacked layers (held by dispersion) tilted by some angle (to allow for multipolar interactions) and with various in-layer separations of molecules (depending on the size of substituents—in our case, larger when the nitro groups are present). Thus, the general scheme of solid state structure of anthraquinone derivatives is fine-tuned by the details of molecular structure. This will be also true for other similar systems, which adds broader perspective to the presented results.

The crystal phase simulations provided a comprehensive description of the solid state structures of the investigated compounds **2** and **3**, in which diverse intra- and intermolecular factors combine to govern the overall self-assembly of the molecular crystals. The dynamics of structural features was investigated, and compound **2** revealed some instances of closer contact between the proton and the acceptor region. The compound **3** exhibits no such events despite more favourable donor–acceptor distance parameters, which should be attributed to the intermolecular contacts between the hydrogen bridges and the neighboring hydroxyl and nitro groups. Finally, the vibrational signatures derived from the CPMD were found consistent with the picture presented by the static models and dynamics. The crystal simulation of **2** showed broadening and red-shift of the $\nu_{OH}$ band, in agreement with the presence of shorter proton–acceptor contacts, increasing the anharmonicity of the proton potential surfaces. 

## Figures and Tables

**Table 1:** Atomic charges values computed based on Atoms in Molecules (AIM) theory at the B3LYP/6-311+G(d) level of theory in vacuo.

Atomic Charge [e]	Compound 1	Compound 2	Compound 3
q$H^{a}$	0.1013	0.5989	0.6022
qO1	—	−1.0945	−1.0869
qC1	−0.0264	0.5807	0.6053
qC2	−0.0369	−0.0332	−0.0199
qC3	0.9535	0.8257	0.8445
qO2	−1.0854	−1.1248	−1.1168
qC4	−0.0369	−0.0332	−0.0199
qC5	−0.0264	0.5813	0.6055
qO3	—	−1.0944	−1.0869
q$H^{b}$	0.1012	0.5988	0.6021

**Table 2:** Bond Critical Points (BCPs) obtained for the selected geometric parameters of compounds **1**, **2** and **3** in vacuo at B3LYP/6-311+G(d) level of theory. Electron density $\rho_{BCP}$ is given in $e\cdot a_{0}^{-3}$ atomic units, and its Laplacian $\nabla^{2}\rho_{BCP}$ in $e\cdot a_{0}^{-5}$ units.

	Compound 1		Compound 2		Compound 3	
BCP	$\rho_{\mathrm{BCP}}$	$\nabla^{2}\rho_{\mathrm{BCP}}$	$\rho_{\mathrm{BCP}}$	$\nabla^{2}\rho_{\mathrm{BCP}}$	$\rho_{\mathrm{BCP}}$	$\nabla^{2}\rho_{\mathrm{BCP}}$
$H^{a}$-C1	0.2818	−0.9690	—	—	—	—
O2-$H^{a}$	—	—	0.0478	0.1559	0.0478	0.1570
$H^{a}$-O1	—	—	0.3320	−2.2345	0.3316	−2.2386
O1-C1	—	—	0.3051	−0.4180	0.3097	−0.4160
C1-C2	0.3068	−0.8498	0.2983	−0.8021	0.2970	−0.7960
C2-C3	02655	−0.6622	0.2794	−0.7247	0.2778	−0.7160
C3-O2	0.4013	−0.1270	0.3675	−0.3033	0.3707	−0.2923
C3-C4	0.2655	−0.6621	0.2794	−0.7247	0.2778	−0.7161
C4-C5	0.3068	−0.8497	0.2983	−0.8021	0.2969	−0.7960
C5-$H^{b}$	0.2818	−0.9690	—	—	—	—
C5-O3	—	—	0.3051	−0.4180	0.3097	−0.4160
O3-$H^{b}$	—	—	0.3320	−2.2345	0.3316	−2.2384
$H^{b}$-O2	—	—	0.0478	0.1559	0.0479	0.1570

**Table 3:** Electron populations of selected basins of the Electron Localization Function related to the intramolecular hydrogen bonds.

Compound	Lp(O2)	Lp(O1)	V(O1-H${}^{a}$)
**1**	2.63	−	−
**2**	2.74	4.32	1.77
**3**	2.74	4.28	1.78

**Table 4:** The results of the SAPT energy partitioning at the SAPT2 level for the dimers of compounds **1**, **2** and **3** depicted in Figure 6. Structures are taken from the crystal X-ray data available [20,21,22]. All energy terms in kcal/mol: Elst—electrostatics; Exch—exchange (Pauli) repulsion; Ind—induction (polarization); Disp—dispersion; SAPT0 and SAPT2 are defined according to Ref. [35].

Dimer Type	Elst	Exch	Ind	Disp	SAPT0	SAPT2
**1a**	−5.48	14.10	−1.47	−19.17	−11.60	−12.01
**1b**	−1.63	2.48	−0.65	−3.38	−3.98	−3.19
**1c**	−2.04	2.10	−0.55	−2.63	−4.08	−3.12
**2a**	−3.10	7.17	−0.78	−9.22	−6.19	−5.93
**2b**	−0.96	2.82	−0.45	−2.68	−0.88	−1.27
**2c**	−1.55	1.76	−0.32	−1.72	−2.06	−1.82
**3a**	−4.62	12.69	−1.73	−16.85	−10.22	−10.51
**3b**	−4.25	5.47	−0.62	−2.53	−2.33	−1.93
**3c**	−2.80	2.65	−0.80	−2.15	−4.11	−3.10

## Data Availability

The data presented in this study are available in the article and in the associated Supplementary Material.

## Supplementary Materials

The following are available online. Figure S1. Molecular forms of **1**—9,10-anthraquinone, **2**—1,8-dihydroxy-9,10-anthraquinone and **3**—1,8-dinitro-4,5-dihydroxy-anthraquinone with atoms numbering scheme. Figure S2. Schematic presentation of the molecule division for the substituent effects discussion. Figure S3. The crystallographic unit cells of 1,8-dihydroxy-9,10-anthraquinone and 1,8-dinitro-4,5-dihydroxy-anthraquinone used to prepare initial models for solid state CPMD simulations. Table S1. Selected geometric parameters of the compound **1**. Comparison of experimental and computed data. Table S2. Selected geometric parameters related to the intramolecular hydrogen bonds and quasi-rings in the compound **2**. Comparison of experimental and computed data. Table S3. Selected geometric parameters related to the intramolecular hydrogen bonds and quasi-rings in the compound **3**. Comparison of experimental and computed data. Table S4. The Ring Critical Points (RCPs) values of the anthraquinone (**1**) and its derivatives (**2**) and (**3**) obtained on the basis of AIM theory at B3LYP/6-311+G(d) level of theory. Figure S4. Electron Localization Function (ELF) isosurface for compounds **1**, **2** and **3**. Figure S5. The O1-H${}^{a}$ group rotation around the C1-O1 bond in the compounds **2** and **3**. Figure S6. Time-evolution of interatomic distances of atoms involved in the intramolecular hydrogen bonds formation of the compound **3**, (a) and (b) results obtained from CPMD in vacuo while (c) and (d) in the crystalline phase.

## Abbreviations

The following abbreviations are used in this manuscript:

NMR	Nuclear Magnetic Resonance spectroscopy
IR	Infrared spectroscopy
DFT	Density Functional Theory
MP2	Møller–Plesset second-order perturbation theory
PES	Potential Energy Surface
AIM	Atoms in Molecules
ELF	Electron Localization Function
SAPT	Symmetry-Adapted Perturbation Theory
TD-DFT	Time-Dependent Density Functional Theory
CPMD	Car–Parrinello Molecular Dynamics
BCP	Bond Critical Point
RCP	Ring Critical Point
ESIPT	Excited-State Intramolecular Proton Transfer
HOMO	Highest Occupied Molecular Orbital
LUMO	Lowest Unoccupied Molecular Orbital
CCDC	Cambridge Crystallographic Data Centre
BSSE	Basis Set Superposition Error
