# Supplementary material for: Competition of Intra- and Intermolecular Forces in Anthraquinone and Its Selected Derivatives
Source: Molecules. 2021 Jun 6;26(11):3448. doi: 10.3390/molecules26113448 (PMC8201066; doi:10.3390/molecules26113448)
Supplement: Supplementary file 1 [file molecules-26-03448-s001.zip › molecules-1240259-supplementary.pdf]

SUPPLEMENTARY INFORMATION

Competition of intra- and intermolecular forces in anthraquinone and its selected derivatives

Kamil Raczyński [1], Andrzej Pihut [1], Jarosław J. Panek [1] and Aneta Jezierska [1]*

[1] University of Wrocław, Faculty of Chemistry, ul. F. Joliot-Curie 14, 50-383 Wrocław, Poland

*Corresponding author: Aneta Jezierska, aneta.jezierska@chem.uni.wroc.pl phone. +48 71 3757 224; fax: +48 71 3282 348

Table of contents:

1. **Figure S1.** Molecular forms of **1** - 9,10-anthraquinone, **2** - 1,8-dihydroxy-9,10-anthraquinone and **3** - 1,8-dinitro-4,5-dihydroxy-anthraquinone obtained at the B3LYP/6-311+G(d,p) level of theory with atoms numbering scheme. The atoms of interest are marked. The dashed line indicates an intramolecular hydrogen bond.
2. **Figure S2.** Schematic presentation of the molecule division for the substituent effects discussion.
3. **Figure S3.** The crystallographic unit cells of (**a**) 1,8-dihydroxy-9,10-anthraquinone and (**b**) 1,8-dinitro-4,5-dihydroxy-anthraquinone used to prepare initial models for solid state CPMD simulations [1,2].
4. **Table S1.** Selected geometric parameters of the compound **1**. Comparison of experimental and computed data. Metric parameters (bond lengths) are given in Å. For atoms numbering scheme see Figure 1SI.
5. **Table S2.** Selected geometric parameters related to the intramolecular hydrogen bonds and *quasi*-rings in the compound **2**. Comparison of experimental and computed data. Metric parameters are given in Å and degrees. For atoms numbering scheme see Figure 1SI.
6. **Table S3.** Selected geometric parameters related to the intramolecular hydrogen bonds and *quasi*-rings in the compound **3**. Comparison of experimental and computed data. Metric parameters are given in Å and degrees. For atoms numbering scheme see Figure 1SI.

7. **Table S4.** The Ring Critical Points (RCPs) values of the anthraquinone (**1**) and its derivatives (**2**) and (**3**) obtained on the basis of AIM theory at the B3LYP/6-311+G(d) level of theory. The electron density ρ_{RCP} is given in $e*a_0^{-3}$ atomic units; its Laplacian ∇^2_{RCP} in in $e*a_0^{-5}$ units. The ring number is indicated by Roman numeral.
8. **Figure S4.** Electron Localization Function (ELF) isosurface for compounds **1 –** (a), **2** – (b) and **3 –** (c). Color coding: green - bond basins, yellow - lone pair basins, blue - hydrogen atom basins and red - core basins.
9. **Figure S5.** The effect of the O1-H^a group rotation around the C1-O1 bond (C2-C1-O1-H^a dihedral angle) on the ground state and two lowest singlet excited states in the compounds a) **2** and b) **3** respectively.
10. **Figure S6.** Time-evolution of interatomic distances of atoms involved in the intramolecular hydrogen bonds formation of the compound **3,** (a) and (b) results obtained from CPMD *in vacuo* while (c) and (d) in the crystalline phase.

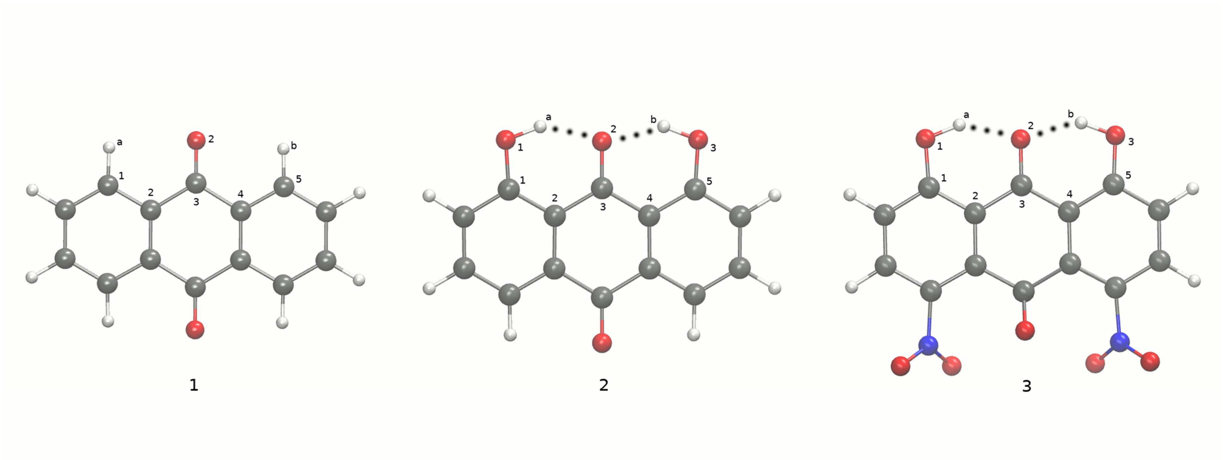

Figure S1. Molecular forms of **1** - 9,10-anthraquinone, **2** - 1,8-dihydroxy-9,10-anthraquinone and **3** - 1,8-dinitro-4,5-dihydroxy-anthraquinone obtained at the B3LYP/6-311+G(d,p) level of theory with atoms numbering scheme. The atoms of interest are marked. The dashed line indicates an intramolecular hydrogen bond.

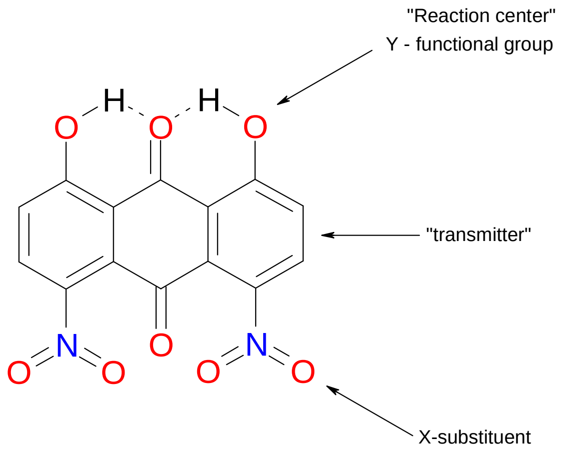

Figure S2. Schematic presentation of the molecule division for the substituent effects discussion.

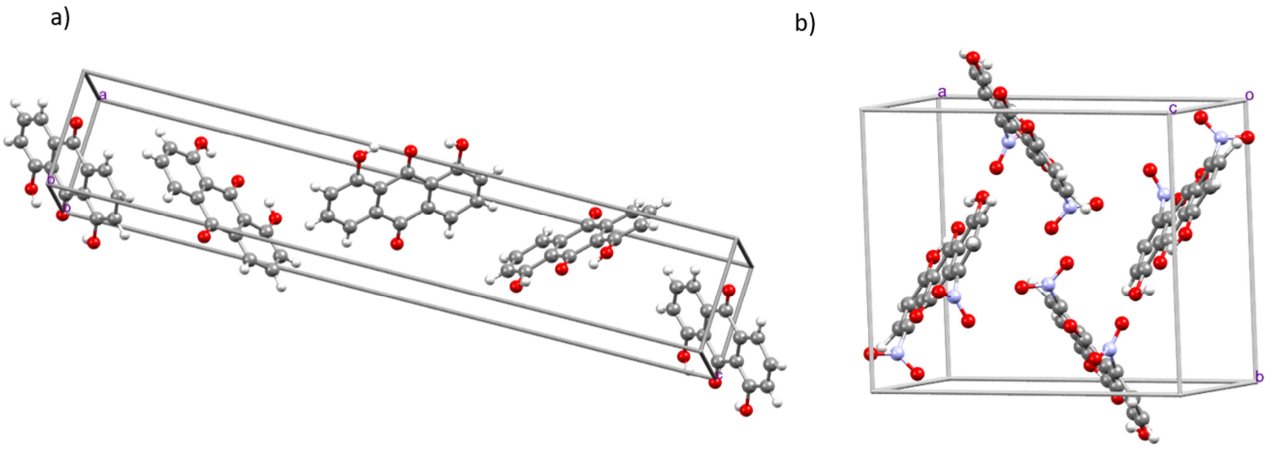

Figure S3. The crystallographic unit cells of (**a**) 1,8-dihydroxy-9,10-anthraquinone and (**b**) 1,8-dinitro-4,5-dihydroxy-anthraquinone used to prepare initial models for solid state CPMD simulations [1,2].

MP2 and DFT geometric parameters of monomers of 9,10-anthraquinone (1), 1,8-dihydroxy-9,10-anthraquinone (2) and 1,8-dinitro-4,5-dihydroxy-anthraquinone (3)

Tables S1-S3 present selected metric parameters obtained as a result of quantum-chemical simulations using MP2 and DFT (B3LYP, PBE, ωB97XD functionals) methods with 6-311+G(d,p) basis set (for clarity see also Figures 1 and S1). The theoretically obtained data was compared with available experimental X-ray measurements [1-3]. The MP2 method served as an additional reference of our theoretical results obtained on the basis of DFT. In case of compound **1** (see Table 1), the H^a-C1, C1-C2, C3-C4 and C5-H^b bond lengths were slightly elongated in all applied methods comparing to the X-ray data. Good agreement with the experimental results was noticed for C2-C3 bond length. However, the C3-O2 bond length was shortened. The H^a-C1 and C5-H^b bond lengths were elongated ca. 0.1 Å with respect to the experimental findings and this is the largest discrepancy noticed comparing the theoretically obtained results with the experimental X-ray data [3]. The compounds **2** and **3** possess intramolecular hydrogen bonds (see Figures 1 and S1). In addition, the compound **3** is substituted in *para* position by NO_2 groups with respect to the OH groups. Therefore, the substituent effects on the hydrogen bonding properties were taken into consideration. The NO_2 group belongs to the group of electron-withdrawing substituents [4]. The intramolecular hydrogen bonds are classified as strong with O…O interatomic distance range between 2.53 Å - 2.57 Å according to the X-ray data [1,2]. There is a competition between the H-bonds, because the acceptor atom (oxygen atom denoted as O2 in Figures 1 and S1) is mutual for both protons in the hydrogen bridges.

The comparison of selected geometric parameters of the compound **2** is presented in Table S2. According to the X-ray data [1] both intramolecular hydrogen bridges are equivalent due to the symmetry exhibited by the compound (therefore the data is reported only for one of the intramolecular hydrogen bonds). The hydrogen bonds belong to the class of Resonance-Assisted Hydrogen Bonds (RAHB) [5]. The presence of the hydrogen bonding resulted in the formation of *quasi*-rings. The O…O interatomic distance of the hydrogen bridge obtained from the computations was slightly elongated comparing to the experimental data [1]. The PBE functional gave the best agreement with the experiment (the O…O distance is 2.558 Å). The O-H bond length given by theoretical approaches is equal ca. 1 Å and comparing to the experimental value of 0.821 Å it is slightly elongated (however it corresponds well with literature references). The H^a…O2 hydrogen bond experimental

distance is 1.834 Å and it is longer comparing to the theoretical results, which range from 1.645 Å for PBE functional to 1.716 Å for ωB97XD. The computed values of the valence angle formed by atoms involved in the intramolecular hydrogen bridge ($O1H^aO2$) are larger comparing to the X-ray data [1]. The largest discrepancy with the experimental measurements was noticed for the data obtained with assistance of the PBE functional. Concerning some bond lengths involved in the anthraquinone moiety (denoted in Figures 1 and S1) we observed that the O1-C1 and C5-O3 bond lengths were shortened comparing to the experimental data, but C3-O2 distance was elongated. Other bond lengths were reproduced with agreement, but again the results obtained with the application of the PBE functional showed the largest discrepancies. However, the observed discrepancies are small enough that we can conclude that the theoretically obtained results correspond very well with the experimental measurements.

The compound **3** has two intramolecular hydrogen bonds, which are not equivalent according to the X-ray data [2]. The computationally obtained results showed that metric parameters of the intramolecular hydrogen bonds are identical (for clarity see Table S3). The O1…O2 and O3…O2 interatomic distances are equal 2.562 Å and 2.570 Å respectively. The difference between them is 0.008 Å. The presence of the NO_2 substituents resulted in the electron coupling in the anthraquinone moiety, which introduced changes in the intramolecular hydrogen bonds properties [2]. The experimentally determined positions of the nitro groups are not equivalent (possibly due to the packing effects), as exhibited by the torsion angles between the nitro group plane and the corresponding aromatic ring: 84.6° and 62.6° for both groups respectively. This introduces not only steric effects, but also different possibilities of resonance coupling between the aromatic system and the nitro groups [2]. The metric parameters differences between unsubstituted anthraquinone and its derivative with OH and NO_2 groups were noticed. The presence of two *quasi*-rings was detected experimentally and based on the computational results. Comparing experimentally measured O…O interatomic distances of the compounds **2** and **3** [1,2], we have noticed that the appearance of the NO_2 groups resulted in the elongation of them. Computationally obtained results (see Table S3) showed that MP2 method gave the largest discrepancies with the experimental findings. The best agreement between theoretical and experimental data was obtained for results derived from simulations with application of B3LYP functional. The results obtained by use of PBE functional showed the shortening of the discussed O…O interatomic distance in both hydrogen bridges. The O-H bond length obtained computationally is equal ca. 1 Å in both hydrogen bonds. However, the experimentally

determined values are for O1-H^a 0.913 Å and for O3-H^b 1.113 Å respectively. The H^a…O2 intramolecular hydrogen bond is equal 1.851 Å according to the X-ray data [2] while the H^b…O2 is equal 1.599 Å. The difference between the geometric parameters is 0.252 Å. Both intramolecular hydrogen bonds present in the molecule are classified as Resonance-Assisted Hydrogen Bonds (RAHB) [5] similarly to compound **2**. The computationally obtained values for H^a…O2 hydrogen bond shortened its length as it is shown in Table S3. The best agreement with the X-ray data was found for MP2 method (the difference is 0.124 Å). In case of the H^b…O2 hydrogen bond, the PBE functional reproduced its geometric parameters most accurately comparing to the X-ray measurements. In the experimental findings, there is a difference between the angle value formed by atoms involved in the intramolecular hydrogen bonds, e.g. the O1H^aO2 angle is equal 133.0° while the O3H^bO2 is equal 142.1°. The applied theoretical approaches provided for the discussed angle value larger than 140°. The results correspond better with the experimental findings for the O3H^bO2 valence angle. The O1-C1 bond length was reproduced correctly, however the MP2 method slightly elongated it while DFT shortened it. The C1-C2 bond length was reproduced correctly, but it is slightly elongated. Similar results were obtained for C3-O2 bond length. The largest discrepancy with the experimental data was noticed for the PBE functional. The C3-C4 bond length was slightly elongated according to MP2, B3LYP and ωB97XD approaches, but the data obtained with assistance of the PBE functional showed its shortening. The C4-C5 bond length was elongated comparing computationally obtained results with experimental measurements while C5-O3 bond length was shortened. The detailed selected geometric parameters analysis showed that the presence of substituents (OH or NO_2 groups) are able to affect slightly the anthraquinone skeleton as it is shown in Tables S2 and S3. It is a valuable qualitative finding concerning the geometry of the studied anthraquinone derivatives. Our computational findings showed that the intramolecular hydrogen bonds are symmetric and equivalent. However, it is visible the NO_2 substituent slight influence on the hydrogen bonding present in the compound **3**. The introduction of additional intramolecular forces in the compounds **2** and **3** resulted in the *quasi*-ring formation and competition between intramolecular hydrogen bonds concerning the interaction with the proton-acceptor oxygen atom. Concluding, our computationally obtained models are in good agreement with experimental data available despite some differences found and described in detail.

Table S1. Selected geometric parameters of the compound **1**. Comparison of experimental and computed data. Metric parameters (bond lengths) are given in Å. For atoms numbering scheme see Figure 1SI.

Metric parameters	***Exp.[3]***	***MP2***	***B3LYP***	***PBE***	***ωB97XD***
**H^a-C1**	0.950	1.086	1.083	1.092	1.083
**C1-C2**	1.367	1.405	1.398	1.407	1.393
**C2-C3**	1.497	1.491	1.492	1.493	1.492
**C3-O2**	1.232	1.228	1.220	1.233	1.212
**C3-C4**	1.472	1.491	1.492	1.494	1.492
**C4-C5**	1.342	1.404	1.398	1.405	1.393
**C5-H^b**	0.950	1.086	1.083	1.092	1.083

Table S2. Selected geometric parameters related to the intramolecular hydrogen bonds and *quasi*-rings in the compound **2**. Comparison of experimental and computed data. Metric parameters are given in Å and degrees. For atoms numbering scheme see Figure 1SI.

Metric parameters	*Exp. [1]*	*MP2*	*B3LYP*	*PBE*	*ωB97XD*
O1…O2	2.533	2.588	2.576	2.558	2.578
O1-H^a	0.821	0.980	0.985	1.005	0.978
H^a…O2	1.834	1.710	1.701	1.645	1.716
<O1H^{a}O2	142.201	147.0	145.7	148.9	144.9
O1-C1	1.361	1.344	1.339	1.341	1.332
C1-C2	1.412	1.420	1.418	1.427	1.411
C2-C3	1.463	1.466	1.461	1.457	1.463
C3-O2	1.225	1.258	1.259	1.279	1.247
C3-C4	1.463	1.466	1.461	1.457	1.463
C4-C5	1.412	1.420	1.418	1.427	1.411
C5-O3	1.361	1.344	1.339	1.341	1.332

*we report on the data for one intramolecular hydrogen bond, because they were indicated equivalent in the crystal structure as well as in the computations.

Table S3. Selected geometric parameters related to the intramolecular hydrogen bonds and *quasi*-rings in the compound **3**. Comparison of experimental and computed data. Metric parameters are given in Å and degrees. For atoms numbering scheme see Figure 1SI.

Metric parameters	*Exp. [2]*	*MP2*	*B3LYP*	*PBE*	*ωB97XD*
O1…O2	2.562	2.599	2.569	2.549	2.576
O1-H^a	0.913	0.979	0.985	1.005	0.978
H^a…O2	1.851	1.727	1.699	1.638	1.718
<O1H^aO2	133.0	146.2	145.0	148.3	144.2
O3…O2	2.570	2.598	2.569	2.549	2.576
O3-H^b	1.113	0.979	0.985	1.005	0.978
H^b…O2	1.599	1.727	1.699	1.638	1.718
<O3H^bO2	142.1	146.2	145.0	148.3	144.3
O1-C1	1.338	1.340	1.332	1.336	1.326
C1-C2	1.412	1.418	1.421	1.428	1.414
C2-C3	1.459	1.470	1.464	1.458	1.466
C3-O2	1.242	1.254	1.256	1.277	1.243
C3-C4	1.462	1.470	1.464	1.458	1.466
C4-C5	1.399	1.418	1.421	1.428	1.414
C5-O3	1.350	1.340	1.332	1.336	1.326

Table S4. The Ring Critical Points (RCPs) values of the anthraquinone (**1**) and its derivatives (**2**) and (**3**) obtained on the basis of AIM theory at B3LYP/6-311+G(d) level of theory. The electron density ρ_{RCP} is given in e*a_0^{-3} atomic units; its Laplacian ∇^2_{RCP} in in e*a_0^{-5} units. The ring number is indicated by Roman numeral.

**RCP**	**Compounds**					
	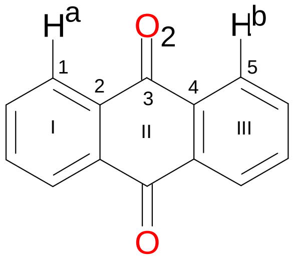		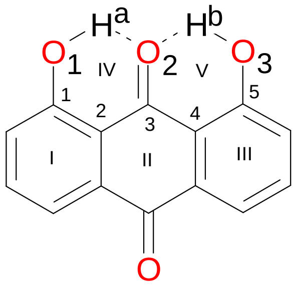		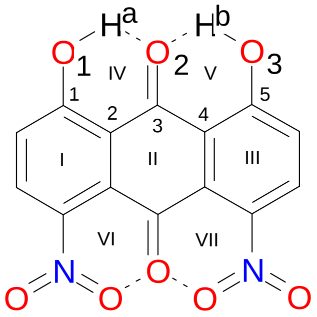	
	ρ_{RCP}	∇^2_{RCP}	ρ_{RCP}	∇^2_{RCP}	ρ_{RCP}	∇^2_{RCP}
**RCP I**	0.0218	0.1594	0.0210	0.1536	0.0210	0.1523
**RCP II**	0.0167	0.1177	0.0170	0.1202	0.0173	0.1219
**RCP III**	0.0218	0.1594	0.0210	0.1536	0.0210	0.1523
**RCP IV**	---	---	0.0185	0.1104	0.0186	0.1109
**RCP V**	---	---	0.0185	0.1104	0.0186	0.1109
**RCP VI**	---	---	---	---	**0.0114***	**0.0570***
**RCP VII**	---	---	---	---	**0.0114***	**0.0570***

*The AIM molecular graph of the compound **3** shows seven RCPs. However, two of them (where NO_2 groups are contributing) do not belong to *quasi*-rings and they are a result of electron density distribution driven by steric effects introduced by the substituents.

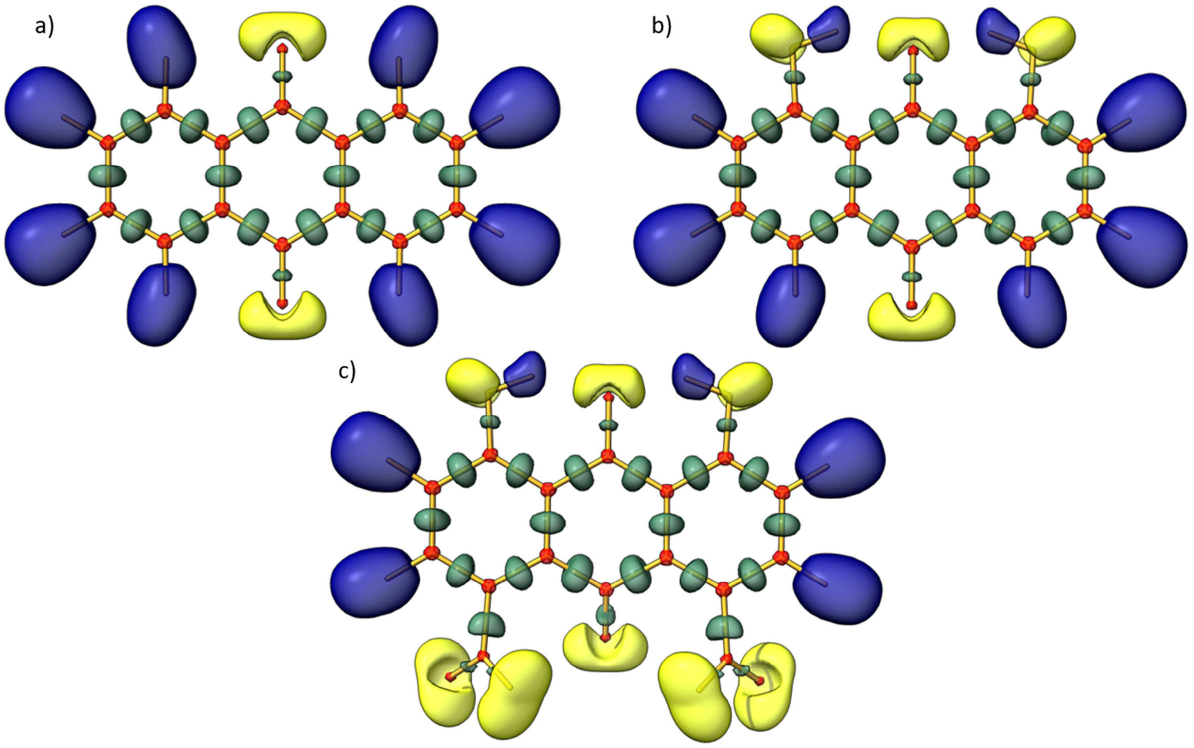

Figure S4. Electron Localization Function (ELF) isosurface for compounds **1** – (a), **2** – (b) and **3** – (c). Color coding: green - bond basins, yellow - lone pair basins, blue - hydrogen atom basins and red - core basins.

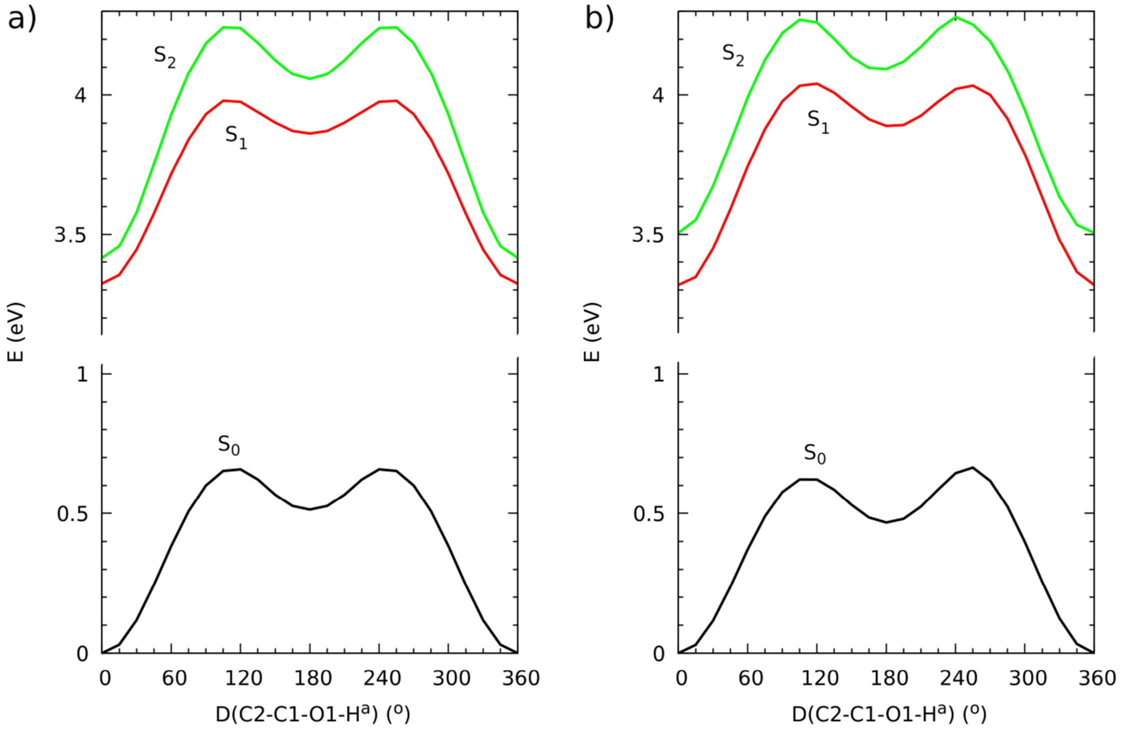

Figure S5. The effect of the O1-H^a group rotation around the C1-O1 bond (C2-C1-O1-H^a dihedral angle) on the ground state and two lowest singlet excited states in the compounds a) **2** and b) **3** respectively.

Car-Parrinello Molecular Dynamics (CPMD) results of intramolecular hydrogen bonds present in the 1,8-dihydroxy-9,10-anthraquinone (2) and 1,8-dinitro-4,5-dihydroxy-anthraquinone (3)

The CPMD simulations were carried out *in vacuo* and in the crystalline phase. The isolated molecule simulations results were compared with the crystalline phase findings to detect differences introduced by various factors, e.g. intermolecular forces and the crystal field. The gas phase simulations of the compound **2** showed that the bridged proton is localized on the donor side in both analyzed intramolecular hydrogen bridges. This is in agreement with our DFT static models findings. The dynamics of both hydrogen bridges is similar as it is shown in Figure 9 (upper part). The average interatomic distances after 70 ps of the CPMD run are: 2.5765 Å for the O1…O2 interatomic distance, 1.0074 Å for O1-H^a bond length and 1.6791 Å for the H^a…O2 hydrogen bond. The average O1…O2 interatomic distance is elongated comparing to the experimental data, but it corresponds well with our MP2 and DFT calculations (see Table S2). The H^a…O2 hydrogen bond length is shorter than that reported by Rohl et al. [1], however it is in agreement with the static models results. Almost identical results were obtained for the O3-H^b…O2 hydrogen bridge. In the crystal structure [1] of the compound **2** various intermolecular interactions are present as it was described in the section 2.3. in the manuscript. The molecules arrangement in the crystallographic unit cell is presented in Figure 2 and Figure S3. The crystalline phase CPMD simulation was performed to include all intermolecular forces influencing the intramolecular hydrogen bonds properties. As it is shown in Figure 9 (lower part) the proton transfer phenomena were not preferable, however short contacts were noticed during 70 ps of the CPMD run. The average values of the hydrogen bridge are as follow: O1…O2 interatomic distance is equal 2.5719 Å, O1-H^a bond length is 1.0108 Å and H^a…O2 hydrogen bond is 1.6906 Å. The O1…O2 interatomic distance is elongated comparing to the X-ray data [1] and it corresponds well with the gas phase findings. However, the hydrogen bond length was shortened and it is closer to the values obtained for gas phase models. The crystalline phase results indicated that the bridged protons were more labile during the CPMD run comparing to the gas phase results. Moreover, the observed short contacts showed that the bridged protons were in the middle of the hydrogen bridges (proton-sharing events). There is no visible correlation between the bridged protons movement in the hydrogen bridges. The short contacts visible in Figure 9 showed that intermolecular forces influenced the dynamics of the

intramolecular hydrogen bonds. The effects of the forces are subtle however they were detected and described quantitatively and qualitatively.

The CPMD results of the compound **3** are presented in Figure S6. The results obtained for the gas phase simulations are in agreement with our MP2 and DFT models. The bridged proton for the whole simulation time is localized on the donor side. The average distances for the atoms involved in the intramolecular hydrogen bonds are: O1…O2 is 2.5713 Å, O1-H^a is 1.0072 Å and for the hydrogen bond 1.6792 Å. The O…O interatomic distance is slightly elongated comparing to the experimental data [2], however the value obtained for the hydrogen bond is smaller than the experimental one and its better corresponds with the MP2 and DFT findings. Similarly to the compound **2**, there is no correlation in the bridged protons dynamics. In the crystalline phase, the bridged proton is also localized on the donor side (see Figure S6 lower part). The crystallographic unit cell with molecules arrangement is shown in Figure 2 and Figure S3. There is an interaction between an oxygen atom from the NO_2 group of neighbouring molecule with the bridged proton. Therefore, there is a competition between intra- and intermolecular forces in the compound **3**. Most probably that is the reason why the bridged proton exhibited a smaller mobility comparing to the compound **2**. The average interatomic distances of the hydrogen bridge obtained from the CPMD run are 2.5571 Å for O1…O2, 1.0111 Å for O1-H^a and 1.6638 Å for the hydrogen bond. Comparing our solid state results with the X-ray data [2] the O…O distance and hydrogen bond length were slightly shortened. However, they correspond well with the DFT static models (see Table S3). The application of the CPMD method gave us a deeper insight in the hydrogen bonds properties as well as indicated the external forces influence on the molecular properties in the investigated anthraquinone derivatives.

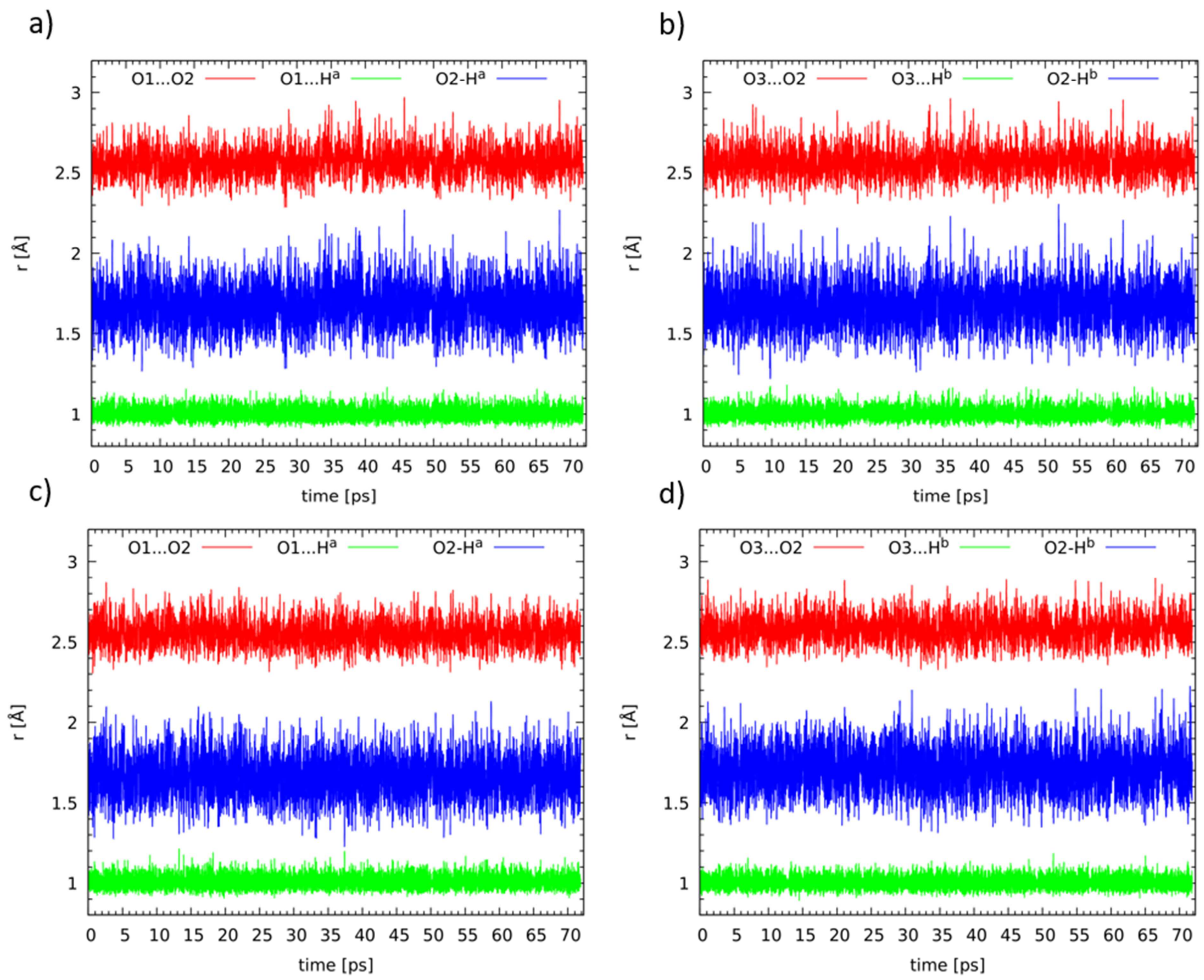

Figure S6. Time-evolution of interatomic distances of atoms involved in the intramolecular hydrogen bonds formation of the compound **3,** (a) and (b) results obtained from CPMD *in vacuo* while (c) and (d) in the crystalline phase.

References:

[1] Rohl, A.L.; Moret, M.; Kaminsky, W.; Claborn, K.; McKinnon, J.J.; Kahr, B. Hirshfeld Surfaces Identify Inadequacies in Computations of Intermolecular Interactions in Crystals: Pentamorphic 1, 8-Dihydroxyanthraquinone. *Crystal Growth & Design*. **2008**, *8*, 4517-4525. 10.1021/cg8005212

[2] Brown, C.J.; Colclough, M.L. 1,8-Dinitro-4,5-dihydroxyanthraquinone, *C14H6N2O8. *Acta Cryst.* **1983**, C*39*, 300-302.

[3] Chen, Y.-J.; Yang, S.-C.; Tsai,C.-C.; Chang,K.-C.; Chuang, W.-H.; Chu, W.-L.; Kovalev, V.; Chung, W.-S. Anthryl-1,2,4-oxadiazole-Substituted Calix[4]arenes as Highly Selective Fluorescent Chemodosimeters for Fe^{3+}. *Chem. Asian J.* **2015**, *10*, 1025 – 1034.

[4] Hansch, C.; Leo, A.; Taft, R.W. A survey of Hammett substituent constants and resonance and field parameters. *Chem. Rev*. **1991**, *91*, 165-195. 10.1021/cr00002a004

[5] Gilli, G.; Bellucci, F.; Ferretti, V.; Bertolasi, V. Evidence for resonance-assisted hydrogen bonding from crystal-structure correlations on the enol form of the .beta.-diketone fragment. *J. Am. Chem. Soc.* **1989**, *111*, 1023-1028. 10.1021/ja00185a035
